# Complete genome sequence of aquaculture pond isolate, *Bacillus velezensis* M4019

**DOI:** 10.1128/mra.00644-24

**Published:** 2024-09-19

**Authors:** Po-Sen Lee, Chung-Yi Liou, Yu-Che Chiu, Cheng-Yu Ma, Po-Wei Huang, Liang-Chun Wang

**Affiliations:** 1 Marine and Pathogenic Microbiology Laboratory, Department of Marine Biotechnology and Resources, College of Marine Science, National Sun Yat-sen University, Kaohsiung City, Taiwan; 2 Committee of Fisheries Extension Service, College of Marine Science, National Sun Yat-sen University, Kaohsiung City, Taiwan; 3 Kaohsiung City Animal Protection Office, Kaohsiung City, Taiwan; 4 Division of Urology, Department of Surgery, Zuoying Armed Forces General Hospital, Kaohsiung City, Taiwan; DOE Joint Genome Institute, Berkeley, California, USA

**Keywords:** *Bacillus velezensis*, aquaculture

## Abstract

*Bacillus velezensis* is commonly found in soil and has various antibacterial activities against animal and plant pathogens. Here, we present the complete genome sequence of *Bacillus velezensis* strain M4019, isolated from a euryhaline aquaculture pond water in Yong-An, Kaohsiung City, Taiwan. This pond-water-derived isolate, unlike common soil-derived isolates, may provide potentially different adaptations and antimicrobial cues for future research.

## ANNOUNCEMENT

*Bacillus velezensis* is a Gram-positive, aerobic, endospore-forming bacterium that produces secondary metabolites with various antimicrobial activities (1). Because of the extensive repertoire of antimicrobial properties, *Bacillus velezensis* has been applied in pesticides for plant diseases and antimicrobial research against human pathogens, emphasizing broad antibiotic application (2–7). We isolated a *Bacillus velezensis* strain from aquaculture pond water in Yong-An District, Kaohsiung City, Taiwan, in November 2022, named this strain M4019, and sequenced its complete genome.

Water samples from an aquaculture pond in Yong-An District were collected and streaked on Luria Agar (LA) and incubated at 37℃ for 24 hours. A single *Bacillus*-like colony with an inhibition zone from adjacent bacterial colonies was isolated and cultivated on LA agar at 37°C for 24 hours. The Gentra Puregene Yeast/Bact Kit (Qiagen, Germany) was used to extract DNA from representative colonies isolated. 16S V1–V9 rRNA gene sequencing of at least three representative colonies prior to whole-genome sequencing revealed *Bacillus velezensis* as the potential species.

A single colony of the isolated *Bacillus velezensis* was cultivated on LA agar at 37°C for 24 hours in preparation for subsequent genome sequencing. The same kit was used to extract 28.64 µg of genomic DNA from approximately 10^11^ bacteria, whose number was estimated with plate count, and the DNA was subjected to whole-genome sequencing. Covaris g-TUBE (Covaris, 520079) was used to shear 1 µg genomic DNA, which was purified using AMPure PB beads (PacBio, 100-265-900). Following the manufacturer’s instructions, the sheared and purified DNA fragments above 20,000 bp were selected as a template to prepare the SMRTbell library via SMRTbell Template Prep Kit 2.0 (PacBio, 101-835-100). The inserts were ligated to adapters after damage and end-repair steps.

Genomics BioSci & Tech Co. (Taipei, Taiwan) performed single-molecule real-time (SMRT) sequencing with 100× coverage on a PacBio Sequel sequencer using a SMRT Cell 1M v3 tray with version 3.0 chemistry. The sequel system was used for the primary filtering analysis, and SMRT Link version 9.0 (8) was used for the secondary filtering analysis. The default parameters were applied for both filtering steps with a quality value cutoff of 20.

The sequence library contained 1,943,654 subreads and an average read length of 3,013 bp. These reads were then used for assembling and polishing the genome with hifiasm version 0.15.3 (9) and GCpp version 2.0.2 (https://github.com/PacificBiosciences/gcpp), respectively. The subreads were corrected using canu version 2.1. Circlator version 1.5.5 (10) was used to circularize the genome, and QUAST version 5.0.2 (11) evaluated the assembled genome quality. The *Bacillus velezensis* genome comprises 3,929,695 bp in one contig, with a GC content of 46.5%. It contains 3,946 predicted genes, of which 3,752 are protein-coding sequences. A total of 86 tRNAs and 27 rRNA operons were predicted using Prokka version 1.14.6 (12). The final closed-circle version of the *Bacillus velezensis* M4019 genome sequence was submitted to GenBank. Default parameters were used except where otherwise noted.

Further studies of the genome of this pond-water-isolated *Bacillus velezensis* M4019 and antimicrobial experiments are of interest and may reveal more information on potential antibacterial activity with aquatic and/or non-aquatic bacterial pathogens.

## Data Availability

The complete genome sequence of *Bacillus velezensis* M4019 was deposited in GenBank under the accession number CP151288. The raw sequencing reads were deposited in the NCBI Sequence Read Archive (SRA) under accession number SRR28713005.
